# High temperature *in situ* gas analysis for identifying degradation mechanisms of lithium-ion batteries†

**DOI:** 10.1039/d4sc08105f

**Published:** 2025-02-12

**Authors:** Leon Schmidt, Kie Hankins, Lars Bläubaum, Michail Gerasimov, Ulrike Krewer

**Affiliations:** a Institute for Applied Materials – Electrochemical Technologies, Karlsruhe Institute of Technology Adenauerring 20b Karlsruhe Germany ulrike.krewer@kit.edu

## Abstract

The primary safety concern associated with lithium-ion batteries is the risk of thermal runaway. The components of the cells can react under heat release when exposed to external or internal heat sources, potentially leading to large-scale fires and explosions. This process is initiated by the decomposition and/or reformation of the Solid Electrolyte Interphase (SEI) and electrolyte; the precise underlying reaction network remains unclear due to insufficient availability of *in situ* chemical analysis methods during thermal abuse. Herein, we present a method based on high-temperature feasible online electrochemical mass spectrometry that is used to investigate these mechanisms and propose a reaction network of SEI formation and degradation. For a graphite/NMC cell with ethylene carbonate/dimethyl carbonate/LiPF_6_ electrolyte, added vinylene carbonate concentration and formation current are shown to impact the composition of the SEI both before and during the thermal stress test up to 132 °C. Higher amounts of the additive vinylene carbonate suppress the evolution of C_2_H_4_ during thermal abuse, suggesting a reduced presence of the organic SEI component lithium ethylene dicarbonate. Our results indicate that the conductive salt decomposition is amplified by the amount of lithium carbonate and reduced by lithium ethylene glycol. This connects the presence of certain SEI compounds directly to the formation of hazardous species. The work highlights the importance of identifying the underlying degradation pathways and for the understanding of the processes that give rise to thermal runaway.

## Abstract 2

This study uses online electrochemical mass spectrometry to track the gassing behavior of carbonate-based electrolyte mixtures in lithium-ion batteries, uncovering key thermal degradation processes.

## Graphical abstract

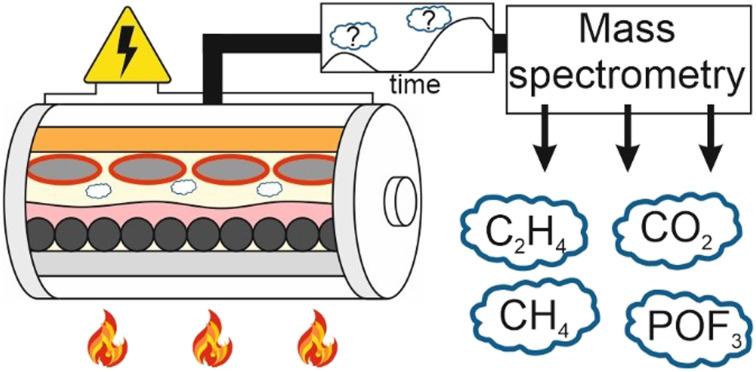

## 1 Introduction

The prevalence of lithium-ion batteries (LIBs) is constantly increasing in the general public due to the growing popularity of electric vehicles and renewable energy resources. This has created a corresponding growth in the concern about battery safety, particularly in regards to thermal stability. Several events in the past showed the hazardous effects of battery fires caused by cell failure.^1,2^ When LIBs are abused mechanically, *e.g. via* deformation, electrically, *e.g. via* overcharge, or thermally, by heating above the permitted temperature, exothermic degradation reactions within the battery can occur. These cause the cell to heat, leading to further and accelerated degradation reactions, which initiate a feedback loop of uncontrolled self-heating leading to thermal runaway.^3–5^

Prior works have outlined and summarized the underlying mechanisms of the heat evolution inside the battery.^1,2,4,5^ The initial reactions that occur at temperatures of approximately 60 °C to 80 °C have been attributed to the decomposition of the conductive salt and the degradation and reformation of the Solid Electrolyte Interphase (SEI).^6–8^ The SEI is a protective layer formed by electrolyte reduction resulting in the production of *e.g.*, solid organic or inorganic lithium salts and polymers on the surface of the negative graphite electrode as well as gaseous species.

The chemical, structural, and performance characteristics of the SEI are strongly affected by the composition of the electrolyte and electrode surface, as well as by formation parameters such as current density and temperature. SEI properties play a substantial role in cell performance, durability, and thermal stability.^5,9^ The elevated temperatures associated with thermal abuse have been shown to trigger reactions that alter and degrade the SEI, leading to cell instability and additional gas production.^10^

The identification of SEI compounds has been the focus of many prior studies, including analytical techniques,^9,11^*ab initio*,^12,13^ and kinetic Monte Carlo simulations.^14^ In order to optimize the SEI composition for increased stability and performance, numerous different strategies have been explored. Two approaches are most common: the use of chemical additives in the electrolyte,^9,15,16^ and the modification of operating conditions during the first formation.^17–19^ The first formation process is often performed with low C-rates, which require long storage times and thus significantly increase production costs.^20,21^ Fast formation may cause lithium-plating during formation,^17^ which increases the probability of thermal runaway during abuse in the cells later life.^22^ Additionally, analysis of the gas evolution during formation revealed that the amount of released CO and C_2_H_4_ increased drastically for fast formation rates indicating increased electrolyte decomposition.^17,18^

A frequently used electrolyte additive for improved SEI formation is vinylene carbonate (VC).^9,23^ Reported positive effects of VC include increased coulombic efficiency during cycling,^24^ higher cyclic and calendaric durability,^25,26^ the suppression of unfavorable side reactions,^27^ and decreased SEI thickness.^28^ Additionally, the thermal stability of the SEI increases when VC is used as an additive.^15,16,29,30^ VC is known to strongly increase the presence of polymers in the SEI.^15,31^ In spite of more than two decades of discussion, the specific polymerization mechanisms and products are unclear.^11,13,32–36^ Recent studies by Lundström *et al.*^35^ revealed the interplay between the commonly used solvent ethylene carbonate (EC) and VC during SEI formation, showing that VC-related reactions are dominant in the presence of both carbonates. Gogoi *et al.*^36^ identified that VC undergoes ring-opening during polymerization leading to polycarbonates or poly vinylene glycol, which potentially undergo hydrolysis. Additionally, it was shown that for initial concentrations of 2 wt% to 3 wt% of VC less than 50% of the additive is consumed during formation for common electrolytes.^18,37^

While VC improves the performance of the negative electrode, multiple studies have revealed detrimental effects of VC on the positive electrode for elevated temperatures.^29,38^ It was reported that VC oxidizes at potentials of 4.3 V *vs.* Li/Li^+^ at room temperature,^9,39,40^ but already at 4.0 V for 70 °C.^30^ VC-containing pouch cells exhibited increased swelling and strong CO_2_ evolution during storage at 90 °C; this behavior was not observed for cells with additive-free electrolyte. The observation was attributed to the formation of a thick cathodic interphase with gas evolution as a side product at high temperatures.^29,38^ While the investigations showed gassing which lead to high internal pressures after multiple hours, it remains unclear if the process is accelerated above 90 °C.

Overall, thermal runaway and the effect of certain cell components and parameters on it are complex. Accelerating Rate Calorimetry has been proven to be a powerful tool for the analysis of thermal degradation in full cells by revealing the heat evolution of the cell during thermal abuse.^3,41^ During investigations with varying contents of VC in the electrolyte, no change of thermal properties was found.^41^ In recent publications, the reaction gases after Accelerating Rate Calorimetry tests were additionally analyzed. The studies showed a variety of species like CH_4_, CO, and CO_2_.^22,42^ The disadvantage of coupling Accelerating Rate Calorimetry with gas analytics is that only the final gas composition is analyzed; the relationship between temperature and gas production remains unknown and significant intermediate products might be missed.

In contrast, other studies have focused solely on the identification of degradation pathways of single battery components during thermal abuse. They identified that the conductive salt LiPF_6_ degrades in the presence of water^8,43,44^ or carbonates,^10^ resulting in the release of HF and POF_3_, which further degrade other cell components. Additionally, other studies focused on the degradation of the SEI and lithiated graphite, identifying interphase changes.^45,46^ The evolution of different degradation products was reported for high and low lithiation degrees of graphite with a SEI during heating.^46–49^ These investigations provided insights about reaction products of isolated cell components, but the complex interplay of all materials in real battery applications cannot be determined. Additionally, information about electrochemical changes including voltage change due to side reactions, as well as reaction products of crosstalk interactions, cannot be observed.

*In situ* methods like Differential/Online Electrochemical Mass Spectrometry (DEMS/OEMS) enable the analysis of chemical degradation mechanisms based on gas evolution during battery operation.^50^ DEMS/OEMS battery investigations typically use lab-scale cells to identify formation processes,^51^ the corresponding reaction pathways of additives,^28,35,36^ and degradation mechanisms related to impurities^52^ or high voltages,^53,54^ all close to room temperature. DEMS/OEMS identifies the analytes from the gas mixtures based on their *m*/*z* values, which can be interfered with by unknown species; recent studies included gas chromatography to overcome this issue.^54^ Gas chromatography provides better insights about gaseous analytes, but measurements become discontinuous and sudden changes in the formation gases cannot be recorded.

Bläubaum *et al.*^55^ introduced a High Temperature – OEMS (HT-OEMS) setup which can measure gas evolution of cells during cycling and thermal abuse up to temperatures of 132 °C. A notable impact of separator materials on thermal stability was shown; studies showed cell voltage, and gas evolution.

In this work, we utilize this HT-OEMS setup to investigate the SEIs' impact on the thermal stability at abuse conditions. The effect of different charging conditions and with different additive concentrations on the SEI formation and cell thermal degradation is studied. The impact of VC and formation current on the thermal degradation is elucidated based on *in situ* gas analysis for the first time, and a reaction network is proposed.

## 2 Materials and methods

This section provides information on the materials and devices used as well as the experiments performed in this study.

### 2.1 Cell assembly

All cells were assembled in a glove box under argon atmosphere (<0.1 ppm O_2_ and <0.1 ppm H_2_O). For electrochemical characterization test cells of the PAT-Series (EL-CELL), and high temperature abuse tests cells similar to the PAT-Series (specifics of the cells used are published by Bläubaum *et al.*^55^) were used. Electrodes with 18 mm diameter (2.54 cm^2^ area) were cut from electrode sheets obtained from CustomCells. Negative electrodes were composed of 96 wt% 260SMG104 graphite active material, with capacities of 2.2 mA h cm^−2^ and 350 mA h g^−1^, styrene-butadiene rubber/carboxymethyl cellulose as binder and a conductive additive on copper foil. Positive electrodes with 2.0 mA h cm^−2^ and 160 mA h g^−1^ consisted of 93.5 wt% lithium nickel (60%) manganese (20%) cobalt (20%) oxide (K-771) active material, polyvinylidene fluoride as binder and conductive additive on aluminum foil. For the calculation of the theoretical capacity of the positive electrodes, every electrode was weighed with a Mettler Toledo XA105DU. Additionally, the uncoated aluminum foil was weighed and the mass of active material was determined by subtraction. Theoretical capacity was determined based on the theoretical gravimetric capacity provided by the manufacturer. The base electrolyte was EC : dimethyl carbonate (DMC), 1 : 1 v/v, 1 M LiPF_6_ mixed by Sigma-Aldrich. Battery grade VC, Sigma-Aldrich, was added to the electrolyte. Commercial cells contain typically 0.5 vol% to 2 vol% of VC: as our cells have a larger volume which needs to be flooded, five times more electrolyte is added compared to commercial formats (calculated from Pritzl *et al.*^40^ with our electrode per electrolyte ratio); to keep the amount of VC constant, VC concentrations of 0.1 vol% and 0.4 vol% were used. As separator a polytetrafluoroethylene membrane, Omnipore JVWP04700, with a porosity of 80% and thickness of 30 μm was used. Lithium metal reference electrodes by EL-CELL, type ECC1-00-0182-O/X, were used.

### 2.2 Electrochemical characterization

Electrochemical characterization was conducted to determine the impact of formation and VC on cell performance at 25 °C using a BaSyTec CTS LAB cycler. After a 4 h resting time after assembly, the cells were charged with either 1C or C/10. The C-rates were based on the theoretical capacity of the positive electrode. Formation included two cycles of constant current (CC) and constant voltage charging (CV) and CC discharging with a upper cutoff voltage of 4.2 V and a lower cutoff voltage of 2.9 V. Cutoff current for the CV-phase was C/20. A relaxation time of 10 min was added between every charging and discharging step. After formation, the nominal discharge capacity was determined for every cell with CC and CV charging and discharging at C/10. The cutoff criteria used for CC and CV were the same as in formation.

### 2.3 High temperature – online electrochemical mass spectrometry

For thermal abuse tests, cells were assembled in the OEMS-PAT-cell in a glovebox and connected to the test stand. The cells and test stand used for this project were described by Bläubaum *et al.*^55^ The setup consists of a Gamry 5000E potentiostat for cycling, Bronkhorst EL-Flow Prestige FG-200CV10 mass flow controllers, a B+B Sensors N480D temperature controller and a Pfeiffer Vacuum GSD320 OC2 mass spectrometer. The carrier gas for the bypass was Argon 5.0 provided by Air Liquide with <2.0 ppm impurities of H_2_O in the gas stream. Before cycling the cells, a leakage check was performed, and the bypass of the cell was flushed with argon for 10 min. The cells then had a 4 h waiting period before the cells were cycled for formation with either 1C or C/10 charging, with the same cutoff criteria as used in the electrochemical cycling. To adjust the time for outgassing of the cell a waiting period of 10 h was added after first charging for 1C formation. The cutoff criterion for discharge was set to 3.0 V. After the formation cycle, cells were charged with CC/CV to 3.7 V, which corresponds to approx. state of charge 50%. The subsequent thermal stress test was performed with a heat rate of 2 °C min^−1^, from room temperature up to a maximum temperature of 132 °C, which is the technical limitation of test setup. The maximum temperature was held for 60 min. After this, heating was stopped and the cell cooled down. Electrodes of selected cells were extracted from the cells after the test, washed with DMC and analyzed under a Keyence VHX7000 light microscope.

For the separation of the signals of C_2_H_4_ and CO during formation, which both have a signal at *m*/*z* 28, a fragmentation relation of C_2_H_4_ between *m*/*z* 26 and *m*/*z* 28 was used.^56^ The residual signal at *m*/*z* 28 is attributed to CO. During thermal stress significant amounts of DMC evaporate, showing fragments with signals at *m*/*z* 2, *m*/*z* 16, *m*/*z* 28, *m*/*z* 44 and others. The contribution of DMC to the signals in relationship to *m*/*z* 90 was measured at 60 °C and subtracted from the total signal to determine the signal of other gaseous species. For *m*/*z* 16 and *m*/*z* 28 DMC contributed most of the recorded signals, making the subtraction sensitive to small deviations.

## 3 Results

This section presents and discusses the electrochemical and mass spectrometry investigations on the impact of VC concentration and formation current on operating and thermal abuse behavior. Insights into performance and gassing during formation and thermal stress are obtained and subsequently used to guide the formulation of hypotheses on the fundamental processes that occur during SEI formation and thermal abuse. The results are used to introduce a reaction network for thermal abuse up to 132 °C. Emphasis is placed on SEI formation and decomposition as well as the impact of VC content.

### 3.1 Performance and gas evolution during formation

The impact of formation current (C/10, 1C) and VC content (0.1 vol%, 0.4 vol%) on the performance of cells at room temperature was investigated with electrochemical measurements and OEMS to assess the cell states before thermal abuse. The results of the electrochemical measurements are summarized in Table 1.

**Table 1:** Formation time, time relation of CC and CV step in 1st charging, coulombic and energy efficiency of first cycle and nominal discharge capacity from a C/10 cycle after formation for the testing parameters

Testing parameter	C/10 with 0.4 vol% VC	1C with 0.4 vol% VC	C/10 with 0.1 vol% VC	1C with 0.1 vol% VC
Formation time 1st & 2nd cycle/h : min	39 : 54 ± 1 : 09	5 : 39 ± 0 : 44	36 : 21 ± 1 : 35	5 : 23 ± 0 : 14
1st cycle coulombic efficiency^a^/%	86.4 ± 0.7	77.8 ± 2.0	81.4 ± 1.4	73.0 ± 1.9
1st cycle energy efficiency^a^/%	85.7 ± 0.9	70.3 ± 2.0	79.6 ± 1.3	64.7 ± 1.8
1st charge CC : CV time relation/% : %	100.0 : 0.0 ± 0.0	43.8 : 56.2 ± 24.2	99.6 : 0.4 ± 0.65	22.4 : 77.6 ± 8.4
Nominal capacity at C/10/mA h g^−1^	163.1 ± 3.3	163.9 ± 1.7	150.4 ± 6.7	157.0 ± 0.7

a When comparing efficiency values of different C-rates it is important to consider that there was no constant voltage step for the discharge cycle. Based on the higher overpotential during 1C fast charge, the cutoff voltage was reached with less charge.

We observed an increased formation time with higher VC content for both, fast and slow formation. These systems also exhibited an increased coulombic efficiency for the first cycle, which is in agreement with prior studies.^24^ We suggest lower loss of lithium inventory and capacity fade to increased amounts of non-lithium-based polymers in SEI for high VC concentration.

It was observed that the formation time reduces by approximately 7 times when applying 10 times higher current densities. For high VC-content, similar nominal capacities for the two formation rates were observed. This indicates no additional loss in lithium inventory with fast formation. The results, shown in Table 1, align with the studies of Münster *et al.*^17^ and Leißing *et al.*^18^ with similar cell components and same VC-content.

**Fig. 1:**
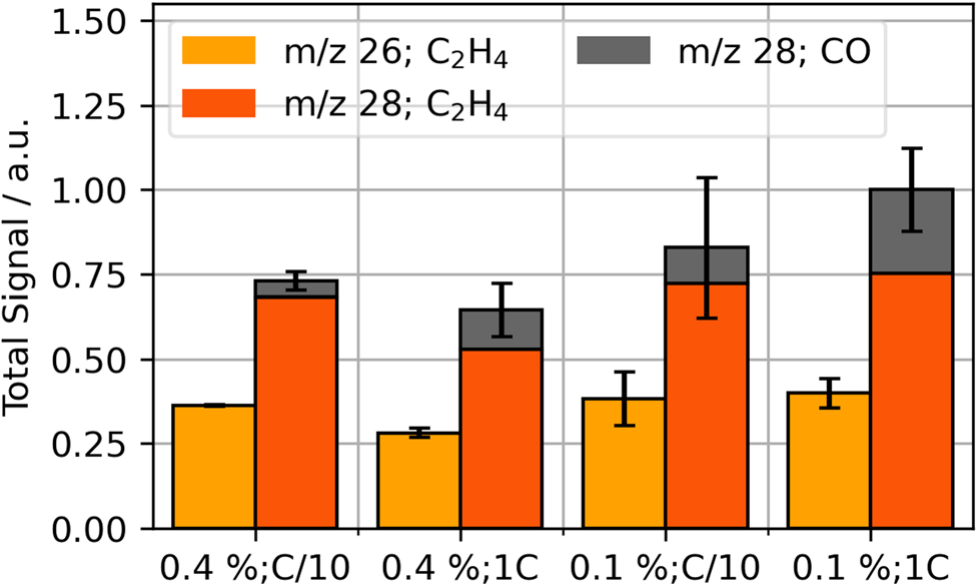
Integrated signal of detected gases CO (*m*/*z* 28, grey) and C_2_H_4_ (*m*/*z* 26, yellow; *m*/*z* 28, orange) from the start of the formation process to 12 hours for various VC concentrations (vol%) and formation currents.

OEMS analysis was performed during the first cycle in order to obtain insights on formation products. The two main signals observed were *m*/*z* 26 (C_2_H_4_) and *m*/*z* 28 (C_2_H_4_ and CO), whereas smaller signals of *m*/*z* 2 (H_2_), *m*/*z* 16 (CH_4_) and *m*/*z* 44 (CO_2_) were observed. The integrated signal for the dominant gases at *m*/*z* 26 and *m*/*z* 28 is shown in Fig. 1.

The gas evolution during formation suggests an impact of VC concentration and formation rate on the total quantities formed: CO fractions increase for fast formation rates and decrease for higher VC concentrations. Additionally, total C_2_H_4_ evolution is reduced for higher concentrations of VC and fast formation. Similar gas compositions were also reported in literature.^15,18^

Leißing *et al.* showed in their studies with deuterium and C^13^ isotope-labeling that the formation gases C_2_H_4_ and CO are mainly generated by the decomposition of EC; DMC can be another source for CO evolution, but it only seems to have a smaller contribution in EC/DMC mixtures.^57^ The most common reaction pathways in literature for generation are summarized in eqn (1)–(3). They produce the SEI species lithium carbonate (Li_2_CO_3_), lithium ethylene dicarbonate (LEDC, (CH_2_OCO_2_Li)_2_) and lithium ethylene glycol (LEG, (CH_2_OLi)_2_).^35,51,57,58^

1 EC + 2Li^+^ + 2e^−^ → Li_2_CO_3_ + C_2_H_4_↑

2 2EC + 2Li^+^ + 2e^−^ → (CH_2_OCO_2_Li)_2_ + C_2_H_4_↑

3 EC + 2Li^+^ + 2e^−^ → (CH_2_OLi)_2_ + CO↑

Literature suggests that, with higher formation currents, electrode surface potentials increase more rapidly which impacts the presence of different SEI-species.^14,35,59^ Additionally, VC impacts the selectivity of SEI formation processes and thus influences the composition of the SEI.^9,35^ Our cells with lower VC content and higher formation rates lead to an increased presence of CO, which suggests higher presence of LEG in the SEI after eqn (3). For high VC contents with low formation rate, the relative amount of gas produced shifts towards C_2_H_4_ compared to the other parameter combinations. This suggests that these system conditions increase the selectivity of EC reduction towards either Li_2_CO_3_ or LEDC.

In this study, we observed no significant presence of CO_2_ (*m*/*z* 44) during formation, similar to the study of Leißing *et al.* with similar electrolyte and materials.^18^ Mechanisms proposed in literature for VC-based SEI-formation suggest CO_2_ production to occur only during the initial step of the polymer formation.^11,13,33^ The corresponding insignificant presence and possible reduction of CO_2_ can further explain the low detection of the gas.^35^

We conclude that variation of formation rate and VC concentration lead to different SEIs. Different SEI composition in turn was suggested in a modeling study to impact thermal abuse behavior.^5^ This is analyzed experimentally in the following.

### 3.2 Accumulated gas evolution during thermal stress test

**Fig. 2:**
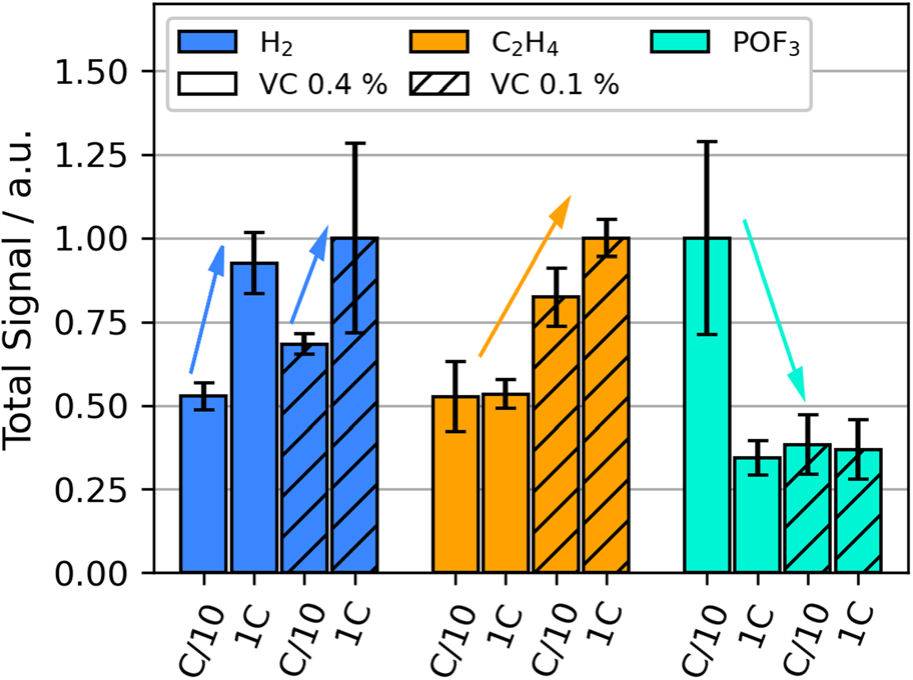
Selected integrated gas signals during the heat-up phase without holding time during the thermal stress test up to 132 °C at SOC 50 for various VC concentrations (vol%) and formation currents: H_2_ (*m*/*z* 2, blue), C_2_H_4_ (*m*/*z* 26, yellow) and POF_3_ (*m*/*z* 104, cyan), average signal intensity for every investigated analyte normalized to 1.

We performed OEMS during temperature stress test in order to elucidate the thermal decomposition reactions in LIBs up to 132 °C. Compared to formation, the diversity of detected signals increased significantly during thermal decomposition: they include significant contributions of *m*/*z* 2 (H_2_), *m*/*z* 26 (C_2_H_4_), *m*/*z* 28 (C_2_H_4_, CO and CO_2_), *m*/*z* 44 (CO_2_) and *m*/*z* 104 (POF_3_), similar as observed in our previous HT-OEMS study.^55^ The impact of formation current and VC content on the amounts of gases with *m*/*z* 2, *m*/*z* 26, and *m*/*z* 104 during the heating ramp from 60 °C to 132 °C is shown in Fig. 2.

The detected H_2_ amount during the heating ramp increases for cells with higher formation rates for both VC concentrations. Prior studies suggest that during thermal runaway, H_2_ is generated above 200 °C by the reduction of the CMC binder material often used in graphite electrodes.^60^ This, however, cannot explain the varying amounts observed here at much lower temperatures. Other sources for H_2_ are the reduction of hydroxy groups, *e.g.* from water impurities and products of electrolyte oxidation.^52,53^ These are further explored in the following.

Spotte-Smith *et al.*^10^ suggested that SEI-species containing hydroxy groups may further be reduced to produce H_2_ at high temperatures. It is also likely that a portion of the H_2_ stems from thermal decomposition of intermediate products, by first the chemical formation of species with acidic or hydroxy groups and their subsequent reduction. Lundström *et al.*^51^ showed that water and EC reduction are competitive processes. EC reduction is suggested to be the dominant process at low potentials whereas water reduction starts at higher potentials. Their follow-up studies also suggested that VC polymerization can consume water during formation.^35,36^ While electrolyte oxidation has been observed at high potentials of the positive electrode, notable degradation rates at elevated temperatures of 70 °C indicate a significantly smaller window for the electrochemical stability of electrolytes at elevated temperatures.^30^ The potential of the negative electrode drops faster during high C-rate formation than low C-rate formation. The lower potential possibly shifts the favorability of solvent reduction to become more dominant, lowering the consumption of water in the cell during SEI formation. The thermally driven reduction of this excess water may lead to a contribution of the higher amounts of H_2_ production observed during thermal abuse for cells with fast formation. A more detailed discussion of the suggested underlying reaction mechanisms is giving in a later section. The good flammability and significant combustion heat released by H_2_ suggests that it potentially plays a role in the thermal runaway of batteries.

C_2_H_4_ evolution during the thermal stress test increases significantly for the lower VC concentrations compared to higher concentrations. A high formation rate further increases C_2_H_4_ release. C_2_H_4_ can be generated by the breakdown of organic SEI-compounds (eqn (4)) or by the rebuilding of the SEI after thermal SEI decomposition (eqn (1) and (2)). It has been observed in literature that VC as a formation additive suppresses LEDC formation in the SEI.^31^ The decreased presence of LEDC would correlate to a decrease in the amount of C_2_H_4_ released during thermal decomposition. The results suggest that VC decreases the extend of SEI breakdown below 132 °C by impeding the build-up of the less-stable organic species. Literature reports the exothermic nature of the breakdown of the organic SEI,^4^ which may also lead to lower onset for self heating temperatures of a battery with a highly organic SEI.^5^

4 (CH_2_OCO_2_Li)_2_ + 2Li^+^ + 2e^−^ → 2Li_2_CO_3_ + C_2_H_4_↑

5 2CO_2_ + 2Li^+^ + 2e^−^ → Li_2_CO_3_ + CO↑

6 Li_2_CO_3_ + 2HF → 2LiF + H_2_O + CO_2_↑

7 LiPF_6_ + H_2_O → LiF + POF_3_↑ + 2HF

The gas evolution of POF_3_ is strongly increased for the cells with high VC content and low formation rate. All other combinations of VC content and formation rate exhibited much smaller amounts of POF_3_. POF_3_ is likely formed by the conductive salt decomposition in the electrolyte (eqn (7)) and further causes electrolyte solvent decomposition forming phosphoric acid derivatives.^6,7,61^ Additionally, it increases the presence of HF in the cell, which can lead to SEI decomposition, see *e.g.*eqn (6). A high presence of POF_3_ thus indicates more decomposition of the conductive salt and consequently also of the SEI and solvents. Higher quantities of highly toxic species like HF and some phosphoric acid derivatives significantly increase the safety risk in the case of cell venting. The production of POF_3_ and related chemical interactions will be further discussed in the following section.

CO and CO_2_ are two gases commonly found in high amounts in thermally abused cells;^22^ the relative quantities of CH_4_, CO_2_ and the combined signal of C_2_H_4_ and CO are shown in the ESI.† 1C formation with high VC content show a bit lower CO_2_ evolution; more discussion see next section. Both gases have many possible sources, including, electrolyte reduction/oxidation,^22,28,51,53^ chemical electrolyte decomposition,^8,62,63^ SEI decomposition,^4,22,47,60^ and CO_2_ reduction (eqn (5)). This complexity impedes the assignment of a tentative reaction mechanism to CO and CO_2_ if no correlation to gases can be identified.

**Fig. 3:**
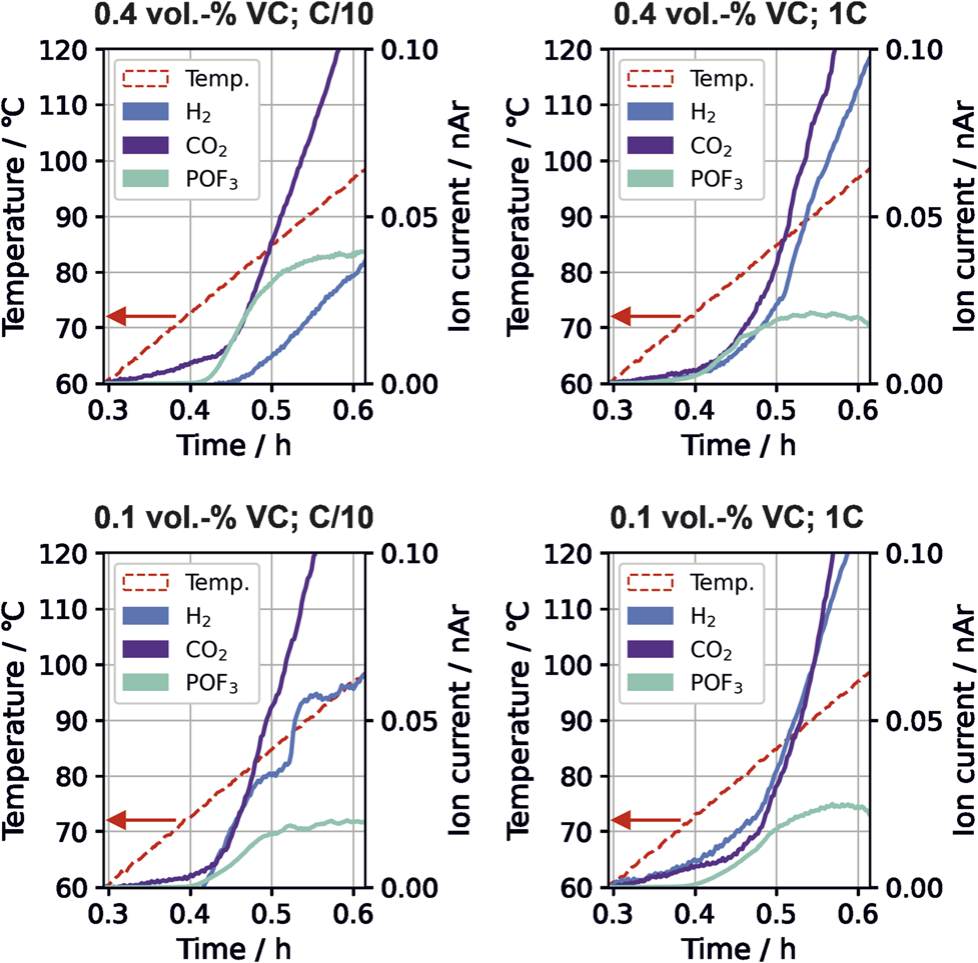
Temperature and gas evolution of H_2_ (*m*/*z* 2, blue), CO_2_ (*m*/*z* 44, purple), and POF_3_ (*m*/*z* 104, cyan) for cells with different VC content and formation rate during the thermal stress test. Signals have been normalized to *m*/*z* 36 (normalized to argon, nAr).

Density functional theory (DFT) calculations (detailed in the ESI†) were performed to determine the likelihood of SEI and salt decomposition reactions as a source of gas evolution and to help delineate the most likely sources of the gases observed with OEMS. The release of Li^+^ and e^−^ from LiC_6_ is known to occur during thermal abuse, which may trigger electrolyte and SEI reduction (eqn (3) and (4)). Gibbs reaction energies, shown in Tables S1 and S2,† reveal that Li released from the anode will favorably react with electrolyte, CO_2_ or organic SEI species to form the gases C_2_H_4_ and CO (eqn (4) and (5)), and that the inorganic species Li_2_CO_3_ will readily decompose to produce CO_2_ in the presence of HF (eqn (6)). CO_2_-forming chemical decompositions of LiPF_6_ and SEI species (Table S2†) are shown to be endergonic and thus energetically unfavorable in the absence of a reduction source or HF. However, sufficiently high temperatures may overcome this, enabling the continued gas production observed in Fig. 3. The decomposition of LiPF_6_ also produces HF (eqn (7)), leading to the initiation of a self-catalyzing cycle with H_2_O reported by Baakes *et al.*^5^ It is important to note that this discussion is based on reaction energies, not reaction barriers; the reactions discussed here may have high barriers that prohibit them occurring at room temperature. More detailed DFT calculations are suggested for future work in order to determine more precise information about the reactivity.

### 3.3 Progression of gas evolution during thermal stress test

Time-resolved gas generation during the thermal stress test was analyzed in order to gain a deeper understanding of the temperature dependence of gas evolution and thus the sequence of degradation reactions. Fig. 3 shows the gas evolution of *m*/*z* 2 (H_2_), *m*/*z* 44 (CO_2_) and *m*/*z* 104 (POF_3_) for the different cells.

For all VC concentrations and formation rates, evolution of CO_2_, POF_3_ and H_2_ started near 70 °C to 80 °C. For high VC concentration with low formation rate, the relative evolved amount of POF_3_ is three times higher than at other conditions (Fig. 2); this is also the only testing parameter set where H_2_ evolution appears significantly later than POF_3_ (Fig. 3). For the other testing parameters, the gases either evolve simultaneously or POF_3_ appears last.

The Li_2_CO_3_ decomposition with HF to form CO_2_ and H_2_O (eqn (6)) and the POF_3_ production from reaction of the conductive salt with water (eqn (7)) build an autocatalytic cycle.^5^ The cycle from H_2_O to HF and back to H_2_O is the driving force for the ongoing conductive salt and Li_2_CO_3_ decomposition as well as CO_2_ and POF_3_ production. Li_2_CO_3_ and LiPF_6_ are available in the cells in large amounts, so a depletion of these species is unlikely during this thermal stress test. The smaller amounts of POF_3_ for three of the testing parameters suggests that this cycle can be disrupted. A possible source for this interruption is another reaction consuming the hydrogen atoms from the cycle.

No H_2_-producing reaction has been previously connected to this cycle. It can be seen from the measurement that increasing amounts of hydrogen evolution coincide with the plateau of POF_3_ production. Here, we suggest the existence of a H_2_-producing reaction that limits the reaction rates within the autocatalytic cycle by consuming some of the required reactant species. This is detailed in the following. The strongly POF_3_-producing cell (C/10, 0.4 vol% VC) exhibited the lowest CO evolution during formation (Fig. 1), which indicates lower amounts of LEG present in the SEI according to eqn (3). Additionally, it was the only one exhibiting first the onset of POF_3_ before H_2_ evolution during the thermal degradation. Combining these observations, we propose a reaction mechanism where LEG acts as a HF scavenger, removing the protons from the autocatalytic cycle and decreases the rate of conductive salt decomposition:

8 (CH_2_OLi)_2_ + 2HF → (CH_2_OH)_2_ + 2LiF

9 (CH_2_OH)_2_ + 2Li^+^ + 2e^−^ → (CH_2_OLi)_2_ + H_2_↑

10 (CH_2_OH)_2_ + *n*EC → H(OC_2_H_4_)_*n*+1_OH + *n*CO_2_

The reaction of LEG with HF to ethylene glycol (EG, (CH_2_OH)_2_) binds the F^−^ anion in LiF (eqn (8)), and the hydroxyl group of the EG is reduced further to form H_2_ (eqn (9)). DFT calculations revealed that these reactions are energetically favorable, shown in Table S3.† Other studies also report that EG tends to polymerize with EC to polyethylene glycol on the negative electrode under CO_2_ evolution (eqn (10)).^28,51,53^ These reactions are able to explain the significantly increased amount of POF_3_ and low amounts of H_2_ with the low presence of LEG in the SEI.

### 3.4 The impact of vinylene carbonate on thermal safety

**Fig. 4:**
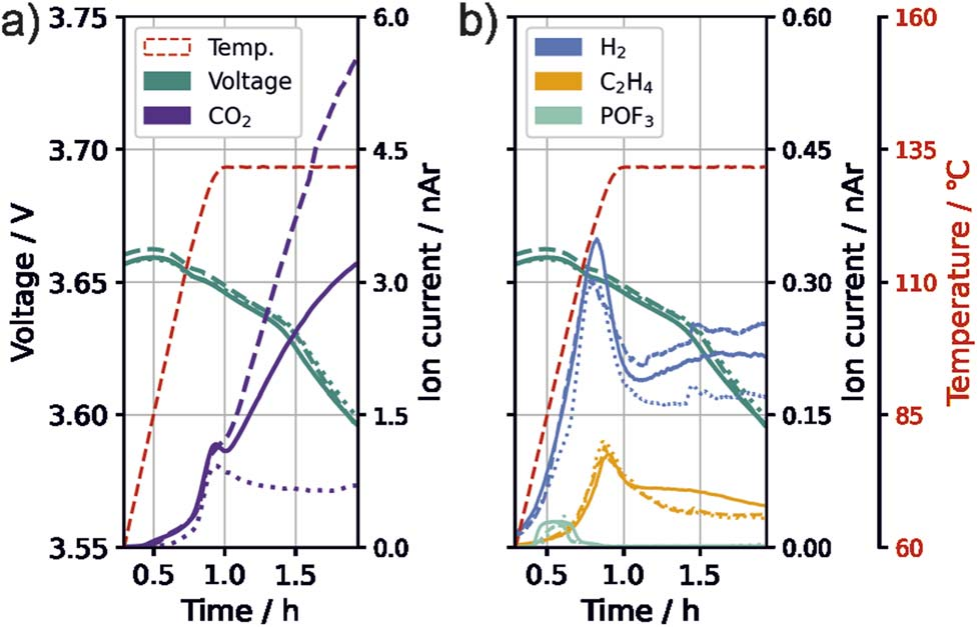
Changes during thermal abuse for three cells (solid, dashed, dotted) with 0.1 vol% VC in electrolyte and a formation rate of 1C. (a) Temperature, open circuit voltage and CO_2_ (*m*/*z* 44, purple). (b) Temperature (red), open circuit voltage (green) and gases with H_2_ (*m*/*z* 2, blue), C_2_H_4_ (*m*/*z* 26, yellow) and POF_3_ (*m*/*z* 104, cyan). Signals have been normalized to *m*/*z* 36 (normalized to argon, nAr).

In the following, we evaluate the 132 °C temperature plateau of the thermal stress tests to gain more mechanistic insights on the effect of VC on thermal safety. We observed high fluctuations in the amount of species with *m*/*z* 44 (likely CO_2_) during the temperature plateau between single test runs. This was most pronounced for cells with 0.1 vol% VC and 1C formation rate. Fig. 4 shows the progression of gas evolution of three test runs for *m*/*z* 44 (CO_2_), *m*/*z* 2 (H_2_), *m*/*z* 26 (C_2_H_4_), and *m*/*z* 104 (POF_3_) as well as of the cell voltage.

Contrary to the behavior during the holding time at 132 °C, all signals, including CO_2_, exhibited a similar trend for all three measured test cells with 0.1 vol% VC and 1C formation during the ramping phase, which is an indication of similar processes occurring within the cells during this stage. During the temperature hold, H_2_ shows a higher intensity when more CO_2_ is detected. C_2_H_4_ and POF_3_ have only minor changes in their intensities. Additionally, the same voltage drop of approximately 60 mV can be observed for all cells. Assuming that the voltage decrease stems from the consumption of lithium from the electrodes or electrode active material loss, similar degree of lithium consumption or active material loss appear for all cells. This indicates that the fluctuating CO_2_ producing reaction is not related to these processes, and is instead likely a reaction of the electrolyte. We thus suggest the existence of a primarily chemical process that sets in at high temperature and produces CO_2_ and H_2_. It is important to note that, due to the test cells used, the electrolyte mass per electrode surface is significantly higher for our cell setup, which could intensify processes of electrolyte degradation.

To get further hints on the origin of the fluctuations, the high-temperature tested electrode surfaces were analyzed visually for solid residuals with a light microscope (ESI Fig. S3†); a new interphase was visible on the positive electrode with strong CO_2_ evolution.

The fluctuation of CO_2_ was not only observed for 0.1 vol% VC and 1C formation, but also for 0.4 vol% VC-content and C/10 formation rate, suggesting that this process is unlikely to be directly influenced by the formation. Fluctuations of this magnitude were not observed for the two other testing parameter combinations within the performed measurements or in our previous study for cells with PTFE separators and without VC.^55^

While literature reports increased SEI thermal stability with VC,^15,16,29,30^ it was observed that cells containing excessive VC exhibit significant gas evolution at high temperature storage.^29,38^ Eom *et al.*^38^ investigated cells with increasing amounts of VC during storage at 90 °C. During post-mortem analysis, they also observed strong increases in the presence of H_2_ and CO_2_ for cells with high VC concentrations in the electrolyte (3–5%). Similarly to our study, they found a new interphase on the positive electrode, which they suggested was a VC-based polymer. However, since in our experiments only 0.1 vol% VC was added, we assume that a film formed solely by VC would be too thin to be visible with a light microscope. This suggests that another species, such as EC, participates in the formation of the observed polymeric surface film.

Polymerizations classically show two steps leading to continuous growth: initiation, and chain-growth reactions. It is likely that VC is involved in the initiation reactions because of its lower electrochemical stability compared to EC. The studies of Lee *et al.*^30^ showed that the oxidation potential of VC is lower than that of EC and is also highly influenced by temperature.

Different initiators for the polymerization of carbonate species have been suggested, including cations,^40,61,64^ PF_5_,^63^ POF_3_ (ref. 44) and hydroxide.^28,51,52^ However, Fig. 4 shows that the evolution of POF_3_ starts at 70 °C and ends before the major CO_2_ evolution starts. Since the polymer was found on the positive electrode, a cation-initiated pathway seems likely. The oxidation pathway of VC suggested by Pritzl *et al.*^40^ can explain the formation of the polymer starter, and the formation of H^+^, both forming CO_2_ evolution. The H^+^ subsequently reacts with the conductive salt, forming HF, which leads to the H_2_ formation observed, *e.g.*, by the SEI degradation pathways suggested in the last section (eqn (8) and (9)).

The chain-growth reaction is likely to be based on a cross-polymerization of VC and EC, which has been previously reported.^34,35^ Other studies revealed that cyclic carbonates are able to rapidly polymerize and evolve CO_2_ from 120 °C.^64,65^ Based on these findings, we suggest a polymerization on the positive electrode, which is initiated by VC. The chain-growth reaction is based on EC and residual VC, causing the ring-opening, followed by decarboxylation at high temperatures.

Based on our current results, we cannot explain the fluctuations within the holding plateau in the H_2_ and especially the CO_2_ formation. On possibility is that the extend of polymerization and CO_2_ evolution is related to stochastic variations in the polymer formation process, which impact the chain growth speed and cut-off reactions. Further investigations to identify the parameter influencing the polymerization trigger and extend are needed.

We draw as a conclusion, that under certain battery conditions, such as exposure to high temperatures, VC and EC containing electrolytes can lead to significant gas evolution and with that can become a potential safety risk. Residual VC can trigger cross-polymerization of EC and residual VC under strong CO_2_ evolution when batteries are abused. Especially, if temperatures of 130 °C are reached, gas evolution may cause a rapid internal pressure increase leading to cell venting. The development of mitigation techniques can be crucial for safe operation.

### 3.5 Formation and degradation mechanism

**Fig. 5:**
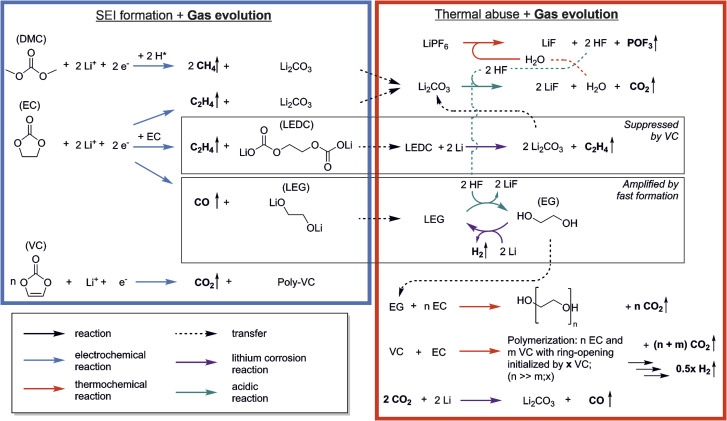
Reaction scheme showing the interplay of reactions occurring during SEI formation (blue box) and thermal abuse (red box) with evolution of gases (marked by vertical arrows).

The reactions that occur during formation are a widely discussed topic, and many pathways and mechanisms have been suggested. However, while a number of reactions have been proposed for formation, there is less research and more uncertainty on which reactions take place at elevated temperatures.^4^ Combined analysis of gas evolution during formation and thermal degradation promises insights into complex inter-dependencies of both and thus to unveil the relationship between the thermal abuse behavior of LIBs and their cell properties and formation conditions. Fig. 5 shows the reactions discussed in the previous sections including the interplay between formation and thermal decomposition processes. Species from formation were connected to thermal decomposition reactions with dotted arrows.

The list includes the most frequently named formation reactions of the following SEI-species: Li_2_CO_3_, LEDC, LEG and poly-VC. Negligible amounts of CO_2_ and CH_4_ were observed during formation. Since literature suggests that VC forms CO_2_ only during the initiation reaction of the polymerization, gas analysis cannot provide information on the propagation, and thus the absolute amount of poly-VC formation in the SEI.^11,35^ The low amount of CO_2_ during formation is attributed to only little occurrence of polymer initiation reaction or to CO_2_ reduction reactions.^35^ However, small amounts of starter reactions can have a major contribution to the SEIs passivation in case of long polymer chains of the poly-VC. The main gases evolving during formation were observed to be C_2_H_4_ and CO. These two gases are often attributed to products of EC reduction, indicating that EC makes a significant contribution to the formation of SEI.

Thermal abuse reactions involve all SEI species except for poly-VC, as well as electrolyte degradation reactions. We suggest different types of reactions which can be driven by either elevated temperature (red arrows), the presence of HF (green arrows), or lithium corrosion (purple arrows). The decomposition of the conductive salt LiPF_6_ starts at 70 °C, and produces the species POF_3_ and HF. Thermally induced reactions involving POF_3_ with electrolyte have been reported in prior thermal degradation studies, including CO_2_ evolution and the formation of phosphoric acid derivatives.^7,43,61^ As phosphoric acid derivatives as well as other unknown analytes cannot be unambiguously identified with OEMS, they are not included, but they can potentially have an impact on all *m*/*z* signals. HF decomposes Li_2_CO_3_, which forms H_2_O, causing the HF/H_2_O cycle which leads to continuous degradation of LiPF_6_ and Li_2_CO_3_.^4,58^ LEDC can be further reduced to form Li_2_CO_3_ and C_2_H_4_, further feeding the auto-catalytic cycle; the formation of LEDC, however, is suppressed in the presence of VC. We propose that the appearance of CH_4_ and C_2_H_4_ is mainly coupled to reformation or alteration of the SEI, causing lithium consumption from the cells inventory and follow the pathway suggested for the formation reactions. LEG can additionally react with HF to form EG, which can either be further reduced to form H_2_ or be polymerized into poly-EG, breaking the auto-catalytic cycle; this pathway is increased in the case of high formation rates.

Several of the reactions discussed above result in CO_2_ production, which can initiate additional reactivity. CO_2_-reduction has been reported by other studies during SEI formation.^35,51^ We suggest that, due to the increased presence of the gas during thermal degradation, CO_2_ leads to the formation of CO and Li_2_CO_3_ (eqn (5)), which in presence of HF will be again decomposed. Lastly we observed strong CO_2_ evolution during the temperature hold, which we associated with a polymerization reactions based on VC and EC.

In other studies, thermal decomposition of LEDC to Li_2_CO_3_, CO_2_, C_2_H_4_ and O_2_ is often suggested.^4,5,22,47^ We did not observe significant evolution of O_2_ in our study, and our DFT calculations also showed that the decomposition of carbonates to elemental oxygen is energetically unfavourable. The electrochemical breakdown of LEDC with additional lithium to form Li_2_CO_3_ and the widely measured C_2_H_4_ (eqn (4)) is more likely as an alternative LEDC decomposition pathway.

We observed that the formation process can significantly impact the prevalence of gases during thermal abuse (see Section 3.2–3.4). The herein presented network can be used to identify reactions pathways based on the gases detected from abused cells in future studies.

## 4 Conclusion

In this study we revealed the impact of formation rate and VC content on gas evolution of LIBs during formation and thermal stress. Based on the composition of the product gases, we suggested degradation reactions for both, formation and thermal abuse, and showed their inter-dependencies. The observed changes in the gas composition during formation indicate that selectivity towards certain SEI products is impacted by both, C-rate and VC content: while a higher formation rate increases the presence of LEG in the SEI, higher VC concentration decreases the presence of this species. During thermal abuse, the first gas evolution reactions begin around 70 °C to 80 °C and comprise mostly the degradation gases POF_3_, CO_2_ and H_2_. Interactions between the decomposition reactions of the conductive salt LiPF_6_, Li_2_CO_3_ and LEG were correlated to gassing during thermal abuse and further validated by DFT calculations. The sensitivity of the ratio of H_2_ and POF_3_ to formation rates and VC concentrations can be explained by a H_2_ producing reaction involving LEG, which suppresses the decomposition cycle of LiPF_6_ and Li_2_CO_3_. This has a direct effect on the quantities toxic species, *e.g.* POF_3_, evolved from the mentioned cycle during thermal abuse of the cells. A large CO_2_ evolution during the cell temperature hold at 132 °C was explained by a polymerization reactions of EC and residual VC, initiated by oxidation of VC. This process possibly can cause cell venting in early stages of thermal runaway.

Our study highlights the effectiveness of HT-OEMS for the investigation of *in situ* gas evolution and the underlying processes during thermal abuse. With this approach, it is possible to access new insights into mechanisms and kinetics of degradation reactions relative to battery composition and SEI state. OEMS may in future be combined with commonly used approaches like Accelerating Rate Calorimetry, to set up and parameterize thermal degradation models like from Baakes *et al.*^4,5^ Such models can be used to develop a better, and especially, quantitative understanding of the reactions, their interactions, and sensitivities to design parameters. The models also allow for developing new knowledge-driven strategies for the mitigation of thermal runaway.

## Author contributions

Leon Schmidt: methodology, formal analysis, investigation, writing – original draft. Kie Hankins: formal analysis, investigation, writing – review & editing. Lars Bläubaum: conceptualization, methodology, writing – review & editing. Michail Gerasimov: conceptualization. Ulrike Krewer: conceptualization, supervision, funding acquisition, writing – review & editing.

## Conflicts of interest

There are no conflicts to declare.

## Supplementary Material

SC-016-D4SC08105F-s001

## Data Availability

Source data are provided with this paper in the KITopen repository under https://doi.org/10.35097/y4fqysd0jspd5qd6.

## References

[cit1] Feng X., Ouyang M., Liu X., Lu L., Xia Y., He X. (2018). Energy Storage Mater..

[cit2] Lyu P., Liu X., Qu J., Zhao J., Huo Y., Qu Z., Rao Z. (2020). Energy Storage Mater..

[cit3] Richard M. N., Dahn J. R. (1999). J. Electrochem. Soc..

[cit4] Baakes F., Lüthe M., Gerasimov M., Laue V., Röder F., Balbuena P. B., Krewer U. (2022). J. Power Sources.

[cit5] Baakes F., Witt D., Krewer U. (2023). Chem. Sci..

[cit6] Kraft V., Weber W., Grützke M., Winter M., Nowak S. (2015). RSC Adv..

[cit7] Kraft V., Weber W., Streipert B., Wagner R., Schultz C., Winter M., Nowak S. (2016). RSC Adv..

[cit8] Solchenbach S., Metzger M., Egawa M., Beyer H., Gasteiger H. A. (2018). J. Electrochem. Soc..

[cit9] Aurbach D., Gamolsky K., Markovsky B., Gofer Y., Schmidt M., Heider U. (2002). Electrochim. Acta.

[cit10] Spotte-Smith E. W. C., Petrocelli T. B., Patel H. D., Blau S. M., Persson K. A. (2023). ACS Energy Lett..

[cit11] Zhang B., Metzger M., Solchenbach S., Payne M., Meini S., Gasteiger H. A., Garsuch A., Lucht B. L. (2015). J. Phys. Chem. C.

[cit12] He X., Bresser D., Passerini S., Baakes F., Krewer U., Lopez J., Mallia C. T., Shao-Horn Y., Cekic-Laskovic I., Wiemers-Meyer S., Soto F. A., Ponce V., Seminario J. M., Balbuena P. B., Jia H., Xu W., Xu Y., Wang C., Horstmann B., Amine R., Su C.-C., Shi J., Amine K., Winter M., Latz A., Kostecki R. (2021). Nat. Rev. Mater..

[cit13] Ushirogata K., Sodeyama K., Okuno Y., Tateyama Y. (2013). J. Am. Chem. Soc..

[cit14] Röder F., Laue V., Krewer U. (2019). Batteries Supercaps.

[cit15] Ota H., Sakata Y., Inoue A., Yamaguchi S. (2004). J. Electrochem. Soc..

[cit16] Ota H., Sakata Y., Otake Y., Shima K., Ue M., Yamaki J.-I. (2004). J. Electrochem. Soc..

[cit17] Münster P., Diehl M., Frerichs J. E., Börner M., Hansen M. R., Winter M., Niehoff P. (2021). J. Power Sources.

[cit18] Leißing M., Horsthemke F., Wiemers-Meyer S., Winter M., Niehoff P., Nowak S. (2021). Batteries Supercaps.

[cit19] Witt D., Bläubaum L., Baakes F., Krewer U. (2024). Batteries Supercaps.

[cit20] Wood D. L., Li J., Daniel C. (2015). J. Power Sources.

[cit21] Liu Y., Zhang R., Wang J., Wang Y. (2021). iScience.

[cit22] Abd-El-Latif A. A., Sichler P., Kasper M., Waldmann T., Wohlfahrt-Mehrens M. (2021). Batteries Supercaps.

[cit23] Burns J. C., Petibon R., Nelson K. J., Sinha N. N., Kassam A., Way B. M., Dahn J. R. (2013). J. Electrochem. Soc..

[cit24] Burns J. C., Sinha N. N., Coyle D. J., Jain G., VanElzen C. M., Lamanna W. M., Xiao A., Scott E., Gardner J. P., Dahn J. R. (2011). J. Electrochem. Soc..

[cit25] Lee H.-H., Wang Y.-Y., Wan C.-C., Yang M.-H., Wu H.-C., Shieh D.-T. (2005). J. Appl. Electrochem..

[cit26] Wu H.-C., Su C.-Y., Shieh D.-T., Yang M.-H., Wu N.-L. (2006). Electrochem. Solid State Lett..

[cit27] Vetter J., Holzapfel M., Wuersig A., Scheifele W., Ufheil J., Novák P. (2006). J. Power Sources.

[cit28] Kitz P. G., Lacey M. J., Novák P., Berg E. J. (2020). J. Power Sources.

[cit29] Jeon J., Yoon S., Park T., Cho J.-J., Kang S., Han Y.-K., Lee H. (2012). J. Mater. Chem..

[cit30] Lee H., Choi S., Choi S., Kim H.-J., Choi Y., Yoon S., Cho J.-J. (2007). Electrochem. Commun..

[cit31] Nie M., Demeaux J., Young B. T., Heskett D. R., Chen Y., Bose A., Woicik J. C., Lucht B. L. (2015). J. Electrochem. Soc..

[cit32] Wang Y., Nakamura S., Tasaki K., Balbuena P. B. (2002). J. Am. Chem. Soc..

[cit33] Michan A. L., Parimalam B. S., Leskes M., Kerber R. N., Yoon T., Grey C. P., Lucht B. L. (2016). Chem. Mater..

[cit34] Kuai D., Balbuena P. B. (2022). ACS Appl. Mater. Interfaces.

[cit35] Lundström R., Gogoi N., Melin T., Berg E. J. (2024). J. Phys. Chem. C.

[cit36] Gogoi N., Lundström R., Hernández G., Berg E. J. (2024). J. Electrochem. Soc..

[cit37] Stockhausen R., Gehrlein L., Müller M., Bergfeldt T., Hofmann A., Müller F. J., Maibach J., Ehrenberg H., Smith A. (2022). J. Power Sources.

[cit38] Eom J.-Y., Jung I.-H., Lee J.-H. (2011). J. Power Sources.

[cit39] Holzapfel M., Jost C., Prodi-Schwab A., Krumeich F., Würsig A., Buqa H., Novák P. (2005). Carbon.

[cit40] Pritzl D., Solchenbach S., Wetjen M., Gasteiger H. A. (2017). J. Electrochem. Soc..

[cit41] Ma L., Xia J., Xia X., Dahn J. R. (2014). J. Electrochem. Soc..

[cit42] Liao Z., Zhang S., Li K., Zhao M., Qiu Z., Han D., Zhang G., Habetler T. G. (2020). J. Energy Storage.

[cit43] Campion C. L., Li W., Euler W. B., Lucht B. L., Ravdel B., DiCarlo J. F., Gitzendanner R., Abraham K. M. (2004). Electrochem. Solid State Lett..

[cit44] Campion C. L., Li W., Lucht B. L. (2005). J. Electrochem. Soc..

[cit45] Parimalam B. S., MacIntosh A. D., Kadam R., Lucht B. L. (2017). J. Phys. Chem. C.

[cit46] Liu X., Yin L., Ren D., Wang L., Ren Y., Xu W., Lapidus S., Wang H., He X., Chen Z., Xu G.-L., Ouyang M., Amine K. (2021). Nat. Commun.

[cit47] Kriston A., Adanouj I., Ruiz V., Pfrang A. (2019). J. Power Sources.

[cit48] Stenzel Y. P., Börner M., Preibisch Y., Winter M., Nowak S. (2019). J. Power Sources.

[cit49] Zhang H., Xue J., Qin Y., Chen J., Wang J., Yu X., Zhang B., Zou Y., Hong Y., Li Z., Qiao Y., Sun S. (2024). Small.

[cit50] Dreyer S. L., Kondrakov A., Janek J., Brezesinski T. (2022). J. Mater. Res..

[cit51] Lundström R., Gogoi N., Hou X., Berg E. J. (2023). J. Electrochem. Soc..

[cit52] Metzger M., Strehle B., Solchenbach S., Gasteiger H. A. (2016). J. Electrochem. Soc..

[cit53] Metzger M., Strehle B., Solchenbach S., Gasteiger H. A. (2016). J. Electrochem. Soc..

[cit54] Zhang H., Wu X., Li Z., Zou Y., Wang J., Yu X., Chen J., Xue J., Zhang B., Tian J., Hong Y., Qiao Y., Sun S. (2024). Adv. Energy Mater..

[cit55] Bläubaum L., Röse P., Baakes F., Krewer U. (2024). Batteries Supercaps.

[cit56] Linstrom P. , NIST Chemistry WebBook, NIST Standard Reference Database 69, 1997

[cit57] Leißing M., Peschel C., Horsthemke F., Wiemers-Meyer S., Winter M., Nowak S. (2021). Batteries Supercaps.

[cit58] Martins M., Haering D., Connell J. G., Wan H., Svane K. L., Genorio B., Farinazzo Bergamo Dias Martins P., Lopes P. P., Gould B., Maglia F., Jung R., Stamenkovic V., Castelli I. E., Markovic N. M., Rossmeisl J., Strmcnik D. (2023). ACS Catal..

[cit59] An S. J., Li J., Daniel C., Mohanty D., Nagpure S., Wood D. L. (2016). Carbon.

[cit60] Golubkov A. W., Fuchs D., Wagner J., Wiltsche H., Stangl C., Fauler G., Voitic G., Thaler A., Hacker V. (2014). RSC Adv..

[cit61] Henschel J., Peschel C., Klein S., Horsthemke F., Winter M., Nowak S. (2020). Angew. Chem., Int. Ed..

[cit62] Guéguen A., Streich D., He M., Mendez M., Chesneau F. F., Novák P., Berg E. J. (2016). J. Electrochem. Soc..

[cit63] Sloop S. E., Kerr J. B., Kinoshita K. (2003). J. Power Sources.

[cit64] Ariga T., Takata T., Endo T. (1997). Macromolecules.

[cit65] Abdul-Karim R., Hameed A., Malik M. I. (2017). RSC Adv..

