# Supplementary material for: High temperature in situ gas analysis for identifying degradation mechanisms of lithium-ion batteries
Source: Chem Sci. 2025 Feb 12;16(12):5118–28. doi: 10.1039/d4sc08105f (PMC11834935; doi:10.1039/d4sc08105f)
Supplement: SC-016-D4SC08105F-s001 [file SC-016-D4SC08105F-s001.pdf]

Supplementary Information (SI) for Chemical Science.
This journal is © The Royal Society of Chemistry 2025

High Temperature in-situ Gas Analysis for identifying degradation mechanisms of Lithium-ion Batteries

Supplementary Information

Leon Schmidt, Kie Hankins, Lars Bläubaum, Michail Gerasimov and Ulrike Krewer

Institute for Applied Materials – Electrochemical Technologies, Karlsruhe Institute of Technology, Adenauerring 20b, 76131, Karlsruhe, Germany

Gas evolution during thermal decomposition

During the in-situ investigations with High-Temperature Online Electrochemical Mass Spectrometry (HT-OEMS), also the gases CH_4 (m/z 16), CO and C_2H_4 (m/z 28), and CO_2 (m/z 44) were observed in mass spectrometry. However, these specific m/z values are highly affected by the solvent dimethyl carbonate (DMC) from the cells electrolyte. Due to high temperatures up to 132 °C solvent evaporation is strongly increased and causes highly intense peaks, i.a. at ratios m/z 16, m/z 28 and m/z 44 fragments of DMC will be observed. Based on the m/z 90 and the relationship between the respective m/z values and m/z 90, we subtracted the contribution of solvent to the signals. For that the ratio of each measurement between the named m/z ratios and m/z 90 was determined at 60 °C and used for subtraction. For m/z 16 and m/z 28 we observed negative signals after the solvent subtraction, which are physically unfeasible. Negative signals were mostly observed during the ramping, when mainly DMC fragmentation contributes to the signals. After the solvent subtraction, only a small signal was left, causing the data processing to be very sensitive to small errors. During the ionization in the mass spectrometer CO_2 fragments also affect m/z 16 and m/z 28, which will increase the detected quantities of the species; in this study these fragments were not subtracted from the shown values, see Figure S1. Additionally, it cannot be excluded that the effect of the changing gas matrix and unknown species in the mass spectrometer over the measurement affects the fragmentation behavior and signal intensity of analytes. The impact of formation current and VC-content on the related gas amounts of CH_4 (m/z 16), CO and C_2H_4 (m/z 28), and CO_2 (m/z 44) during the heating ramp from 60-132 °C are shown in Figure S1.

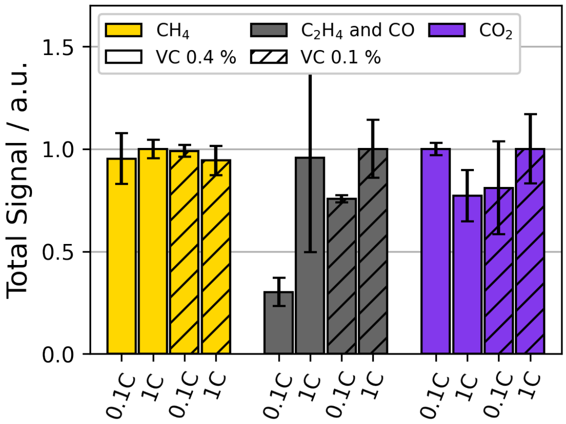

Figure S1: Integrated gas signals during the thermal degradation with temperature up to 132 °C of CH_4 (m/z 16, yellow), CO and C_2H_4 (m/z 28, grey) and CO_2 (m/z 44, violet), signal intensity for every investigated analyte normalized to 1. Solvent subtraction was applied.

Figure S2 shows the repeated measurements from Figure 3 of the main text. The same behaviour discussed in the paper can be observed for the repetitions.

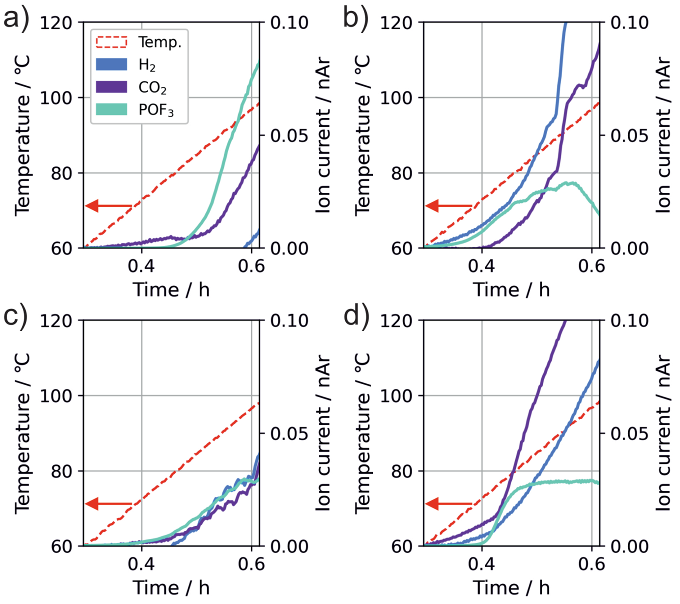

Figure S2: Temperature and gas evolution of H_2 (m/z 2), CO_2 (m/z 44), and POF_3 (m/z 104) during the thermal stress test for different formation rates and VC contents. Signals have been normalized to Argon (nAr, m/z 36). a) 0.4 vol.-% VC with formation rate C/10, b) 0.4 vol.-% VC with formation rate 1C, c) 0.1 vol.-% VC with formation rate C/10, d) 0.1 vol-% VC with formation rate 1C.

Figures S3, S4 and S5 show the behavior of gases CO_2, H_2, C_2H_4, and POF_3 and cell voltages during the thermal ramp for all combinations, except of 0.1 % VC concentration and 1C formation which can be found in the main document.

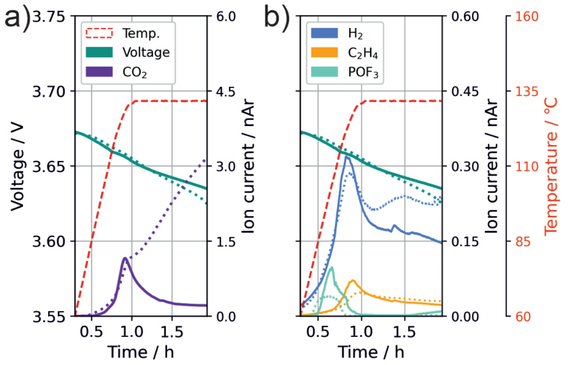

Figure S3: Changes during thermal abuse for two cells (solid, dotted) with 0.4 vol-% VC in electrolyte and a formation rate of C/10. (a) Temperature, open circuit voltage and CO_2 (m/z 44, purple). (b) Temperature (red), open circuit voltage (green) and gases with H_2 (m/z 2, blue), C_2H_4 (m/z 26, yellow) and POF_3 (m/z 104, cyan).

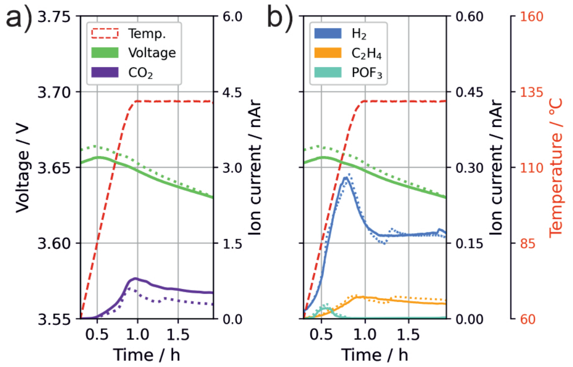

Figure S4: Changes during thermal abuse for two cells (solid, dotted) with 0.4 vol-% VC in electrolyte and a formation rate of 1C. (a) Temperature, open circuit voltage and CO_2 (m/z 44, purple). (b) Temperature (red), open circuit voltage (green) and gases with H_2 (m/z 2, blue), C_2H_4 (m/z 26, yellow) and POF_3 (m/z 104, cyan).

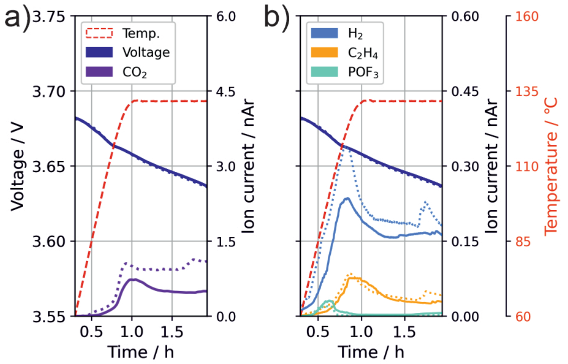

Figure S5: Changes during thermal abuse for two cells (solid, dotted) with 0.1 vol-% VC in electrolyte and a formation rate of C/10. (a) Temperature, open circuit voltage and CO_2 (m/z 44, purple). (b) Temperature (red), open circuit voltage (green) and gases with H_2 (m/z 2, blue), C_2H_4 (m/z 26, yellow) and POF_3 (m/z 104, cyan).

Density Functional Theory Calculations

Density Functional Theory (DFT) calculations were performed using the Gaussian 16 software package [1] in order to obtain reaction energies and develop an initial idea of the possible reactions in the system. All calculations were performed using B3LYP functional with a 6-311++g(d,p) basis set. [2]–[4] The polarized continuum model, [5] with a dielectric constant set to 31.41, corresponding to 1:1 EC:DEC, [6] was used to model the electrolyte environment during reactions.

Table S1: Calculated reaction energies for selected formation reactions addressed in section 3.1

Reactants	Products	Δ G (kcal/mol)
EC + Li^+ + e^-	$LiOCOCH_2CH_2O$	+8.7
$LiOCOCH_2CH_2O$ + Li^+ + e^-	$(CH_2OLi)_2$ + CO	-111.3
EC + $2Li^+$ + $2e^-$	$LiOCOCH_2CH_2OLi$	-95
$LiOCOCH_2CH_2OLi$	$(CH_2OLi)_2$ + CO	-7.6
EC + Li^+ + e^-	$LiCO_3CH_2CH_2$	-51.9
$LiCO_3CH_2CH_2$ + Li^+ + e^-	C_2H_4 + Li_2CO_3	-107.7
EC + $LiOCOCH_2CH_2O$ + Li^+ + e^-	CO + $LiCO_3CH_2CH_2OCH_2CH_2OLi$	-135.8
EC + $LiOCOCH_2CH_2OLi$	CO + $LiCO_3CH_2CH_2OCH_2CH_2OLi$	-32.2
$LiCO_3CH_2CH_2OCH_2CH_2OLi$ + $2Li^+$ + $2e^-$	C_2H_4 + Li_2CO_3 + $(CH_2OLi)_2$	-135.1
EC + $LiOCOCH_2CH_2OLi$ + Li^+ + e^-	CO + $Li_2CO_3CH_2CH_2OCH_2CH_2OLi$	-43
$Li_2CO_3CH_2CH_2OCH_2CH_2OLi$ + Li^+ + e^-	C_2H_4 + Li_2CO_3 + $(CH_2OLi)_2$	-124.3
EC + $LiCO_3CH_2CH_2$ + Li^+ + e^-	$(CH_2OCO_2Li)_2$ + C_2H_4	-117.8
$(CH_2OCO_2Li)_2$ + Li^+ + e^-	Li_2CO_3 + $LiCO_3CH_2CH_2$	-41.8

Table S2: Calculated reaction energies for selected thermal decomposition reactions addressed in section 3.2

Reactants	Products	Δ G (kcal/mol)
$(CH_2OCO_2Li)_2$ + $2Li^+$ + $2e^-$	Li_2CO_3 + C_2H_4	-193.3
Li_2CO_3 + 2HF	2LiF + H_2O + CO_2	-16.3
$LiPF_6$ + H_2O	LiF + POF_3 + 2HF	33.8
Li_2CO_3	CO_2 + Li_2O	63.3
$(CH_2OCO_2Li)_2$	CO_2 + $LiCO_3CH_2CH_2OLi$	18.9
$LiCO_3CH_2CH_2OLi$	CO_2 + $(CH_2OLi)_2$	17.1
$(CH_2OCO_2Li)_2$	$1/2O_2$ + Li_2CO_3 + C_2H_4 + CO_2	50.9
CO_2 + $2Li^+$ + $2e^-$	Li_2O + CO	-109
CO_2 + Li^+ + e^-	$LiCO_2$	-36.5
Li_2CO_3 + Li^+ + e^-	Li_2O + $LiCO_2$	26.8
$LiCO_2$ + Li^+ + e^-	CO +Li_2O	-28.8
CO_2 + $LiCO_2$ + Li^+ + e^-	Li_2CO_3 + CO	-92.1

Table S3: Calculated reaction energies for selected thermal decomposition reactions addressed in section 3.3

Reactants	Products	Δ G (kcal/mol)
$(CH_2OLi)_2$ + 2HF	$(CH_2OH)_2$ + 2LiF	-47.5
$(CH_2OH)_2$ + $2Li^+$ + $2e^-$	$(CH_2OLi)_2$ + H_2	-149.5
$(CH_2OLi)_2$ + HF	$LiOCH_2CH_2OH$ +LiF	-24.7
$LiOCH_2CH_2OH$ +HF	$(CH_2OH)_2$ + LiF	-22.8

Post-mortem analysis with microscopy

After the thermal stress test, cells were opened at air, and the electrode-separator stack was separated. It was observed that after heat-up the electrodes and separator stuck together. After separation, electrodes and separators were washed in DMC and analyzed with the light microscope. Figure S6 shows the positive electrodes after washing with DMC for C/10 (a) and 1C formation (b), for 0.1 vol.-% VC.

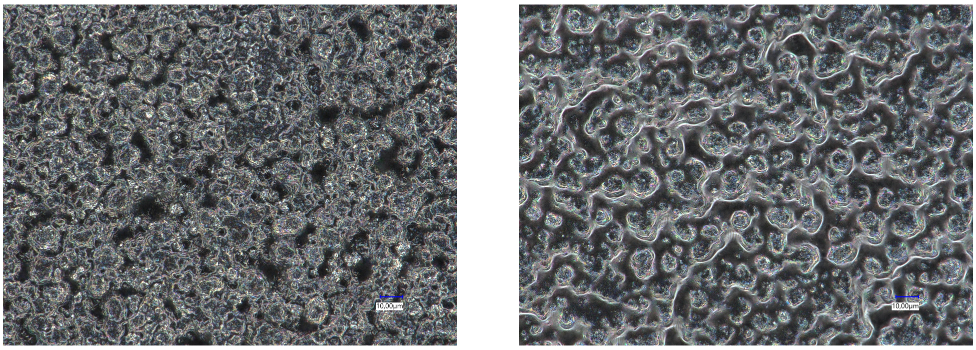

(a) (b)

Figure S6: Light microscope picture of washed NMC 622 electrodes after thermal stress test from cells containing 0.1 vol.-% VC: a) C/10 formation, b) 1C formation.

The test with C/10 formation did not show increased CO_2 evolution, while the 1C formed cell (b) did. Light microscopy pictures reveal significant changes to the surface on b). Due to the increased smoothness of the surface we suggest the presence of an additional film on the positive electrode, indicating solid residuals of a reaction product. Additionally, movement of the bright edges in b) was observed, likely to be due to trapped solvents below the film. We believe that the appearance of the surface film is related to the CO_2 evolution. This is discussed in section 3.4 of the main text.

References

[1] Gaussian~16 Revision C.01, 2016, Gaussian Inc. Wallingford CT.

[2] P. J. Stephens, F. J. Devlin, C. F. Chabalowski, and M. J. Frisch, “Ab Initio Calculation of Vibrational Absorption and Circular Dichroism Spectra Using Density Functional Force Fields,” en, The Journal of Physical Chemistry, vol. 98, no. 45, pp. 11 623–11 627, Nov. 1994.

[3] S. Debnath, V. A. Neufeld, L. D. Jacobson, et al., “Accurate Quantum Chemical Reaction Energies for Lithium-Mediated Electrolyte Decomposition and Evaluation of Density Functional Approximations,” en, J. Phys. Chem. A, vol. 127, no. 44, pp. 9178–9184, Nov. 2023. (visited on 01/21/2025).

[4] A. D. Boese, J. M. L. Martin, and N. C. Handy, “The role of the basis set: Assessing density functional theory,” en, J. Chem. Phys., vol. 119, no. 6, pp. 3005–3014, Aug. 2003. (visited on 01/21/2025).

[5] J. Tomasi, B. Mennucci, and R. Cammi, “Quantum Mechanical Continuum Solvation Models,” en, Chem. Rev., vol. 105, no. 8, pp. 2999–3094, Aug. 2005.

[6] C. Wohlfarth, “Dielectric constant of the mixture (1) ethylene carbonate; (2) dimethyl carbonate,” in Supplement to IV/6, W. Martienssen and M. Lechner, Eds., vol. 17, Series Title: Landolt-Börnstein - Group IV Physical Chemistry, Berlin, Heidelberg: Springer Berlin Heidelberg, 2008, pp. 1033–1033.
